# Hospital and Institutional News

**Published:** 1916-07-08

**Authors:** 


### HOSPITAL AND INSTITUTIONAL NEWS.

### THE ROYAL MEDICAL BENEVOLENT FUND.

The long-established and recognised benevolent society of the medical profession, the Royal Medical Benevolent Fund, has answered many and pressing calls since the outbreak of war, and has many more which it would like to answer were means available. In issuing an appeal for support to wider circles than it has ever approached before, the Fund very justly points out that Territorial medical officers, and others who have joined for service, have had to leave their practices at the shortest notice, and often without being able to make satisfactory arrangements for substitutes. Working expenses of such practices cannot in many cases be materially reduced: rent, rates, and insurance brook no delay, and school fees for children must be paid. The pay of a junior officer in the R.A.M.C. is liberal enough for a young bachelor, just qualified and with no responsibilities; but is not a living wage for men with wives and families, hitherto earning twice or three times as much, and committed to standing expenses of the kind just indicated. There are, of course, even harder cases when the breadwinner has been killed or incapacitated. The Benevolent Fund not only makes grants in such hard cases, but also works in co-operation with the Officers' Families' Association and other agencies. But its resources are practically exhausted, as far as new cases and emergencies are concerned, and the managers are driven to appeal to the public as well as to the medical profession. We trust that from both a liberal response will be forthcoming, and that this excellent Fund will be able to continue to relieve the sad cases which the war is certain to create in the future as in the past; its offices are at 11 Chandos Street, Cavendish Square, W.

### THE TEMPLE FETE.

On the afternoons of July 13 and 14 a fête is to be held in the Middle Temple Hall and Gardens in aid of the British Red Cross and Order of St. John of Jerusalem; a portion of the profits is also being assigned to the Croix Rouge Française. The chief attraction of the fête, though far from the only one, is the performance in the Middle Temple Hall of scenes from "Twelfth Night," first produced in that same hall on February 2, 1601. The production is being organised by Miss Lillah McCarthy, and the prices of admission to it (which include admission to the gardens) range from fifteen shillings to five guineas; two performances will be given each afternoon, owing to the limited seating capacity of the hall. Shakespeare's will is to be on view in the gardens, where also Elizabethan dances, madrigals, and other attractions are arranged for. Given fine weather, the funds of the joint societies should benefit very handsomely: the fourth million has already been passed, but the calls upon the societies grow continually more pressing, and every penny of these enormous sums of money is wisely expended on one or other of the many objects which come within the scope of the splendid work of these societies.

### SIR W. OSLER ON THE WELSH MEDICAL SCHOOL.

A meeting of general practitioners in North Wales, presided over by Dr. Drinkwater, listened to Sir William Osler last week on "The Proposed National Medical School for Wales." Sir William Osler said that a great beginning had been made at Cardiff. The equipment was there, and the great question was the gradual development of those laboratories when the new buildings were occupied. A medical school was so costly to maintain that, considering the size of Wales, and in spite of the sometimes conflicting opinions of men of the North and South in the Principality, he very much doubted if it were worth their while to consider the establishment of more than one medical school. The idea at present was that the heads of the different units should be whole-timers; but whether they had the courage to start on those lines in Cardiff time alone would show. Chemistry would probably show an enormous development in the next twenty-five years; and they in Bangor could help the development of the new school, because the constituent colleges at Bangor and Aberystwyth could easily devise a curriculum specially adapted for intending medical students.

### THE IDEAL DEAN FOR THE WELSH MEDICAL SCHOOL.

Sir William Osler says that, with the support of the Welsh doctors, the proposed National School of Medicine at Cardiff may be made one of the best in the country, if not in the world, and that if the Welsh metropolis rises to the occasion in the matter it will be able to reduce the death-rate from preventible causes by 50 per cent. in five years. As to the head of the new school, Sir William Osler expresses the opinion that the man selected should be comparatively young, well trained, and with a wide outlook; should know a certain amount of science, appreciate a great deal more, and be a good practical man; should love his patients, get on well with his professional brethren, treat his nurses well, and, above all, have sympathy with his students. The ambition of the promoters of the Medical School scheme is to establish an institution of the very first rank, so as to make it worth the while of the northern portion of the Principality to look to Cardiff rather than to the medical schools of Liverpool and Manchester, as at present.

### THE LATE LORD SANDWICH.

The death last week of the Earl of Sandwich removes a genuine personality from the peerage, who in his seventy-six years of life had acquired a reputation for many things, including powers of spiritual healing and treatment by suggestion. Besides being a philanthropist, who established at Huntingdon, near his family seat, a home for boy waifs, he wrote a curious volume entitled "My

Experiences in Spiritual Healing." He gave evidence before the Clerical and Medical Committee of Inquiry into Spiritual Faith and Mental Healing, stating that he would treat cases with or without doctors whenever he was asked to do so. He had had a large experience: a Hindu monk, a Mohammedan, a Hindu princess were among his patients; and he had attended cases in cottages, hospitals, palaces, homes, in a mosque, and in a Hindu monastery. In presiding during 1913 at the opening of a medico-psychological clinic in London for the treatment of disease by psychotherapy he related how after the South African War he had some sixty wounded officers in his country house without the aid of a single doctor or nurse. He expressly disclaimed medical or surgical knowledge, regarded his powers as a divine gift, claimed varied success in most serious diseases, but confessed himself unable to explain his powers. He only knew their potency, and believed that there must be many others endowed with similar powers in a less degree at any rate than himself. His method was that of St. James: prayer and the laying-on of hands, and he said that he acted by intuition. The instances that he gave of his cures included cancer, blindness, paralysis, and sciatica. It was the fourth earl who invented the sandwich as a ready and sustaining meal. The new peer, the nephew of the deceased, is founder of the "Little Commonwealth," a self-governing community of children of both sexes drawn from reformatories, so that the tradition of originality in the family is being maintained.

### HOSPITAL SECRETARY'S SON KILLED.

The sympathy of all institutional workers will go out to the well-known secretary of St. Mary's Hospital, Mr. Thomas Ryan, and Mrs. Ryan, in the death of their son, Captain John Stanley Ryan, who was killed in action in France on June 25. Mr. Ryan had two sons serving; the elder is a lieutenant in the Middlesex Regiment, the second was the gallant officer whose death is announced and who was in the King's Royal Rifle Corps. Captain Ryan, who was aged twenty-six, was educated at Merchant Taylors' School, and became a chartered accountant in the City of London; he was a prominent cricket and football player, and specially distinguished in Rugby football, having played in the International Trials in two recent winters. He joined the Honourable Artillery Company as a private, left for France in September 1914, served for one year and attained the rank of sergeant, when he obtained a commission in the K.R.R.C., being gazetted captain on April 25 last.

### EDUCATIONAL FACILITIES FOR WOMEN STUDENTS IN LONDON HOSPITALS.

In a recent number of the *British Medical Journal* Professor Halliburton, Dean of the Medical Science Faculty of King's College, announces that his school is able to take twenty women students for the preliminary and intermediate portions of the medical curriculum, and that there is little doubt that such students will be able to secure clinical teaching also in time for their final years of medical study. This reform becomes operative next October, and King's thus becomes the third of the old schools in London to throw its doors open to women since the outbreak of war. Professor Halliburton mentioned that St. George's has done this: and the inference, drawn from a condensation of his remarks in the daily Press, that no other school had done the same at once elicited a protest from St. Mary's Hospital, which also admits women students. There are thus now, with the Royal Free Hospital, four general hospitals with schools open to women in the Metropolis.

### A VETERAN OF THE SIKH WARS.

The remaining survivors of the Sikh Wars, than which few more glorious campaigns are inscribed in the annals of the British Army, must be very scanty in numbers now: the oldest of them, and the *doyen* of the medical profession, has just passed away in Adelaide in the person of Surgeon-Major Hinton, at the age of one hundred and three. He was born in 1813, educated at the London and Westminster Hospitals, and qualified in 1835, more than eighty years ago. Gazetted to the Indian Army in 1839, he was present at Aliwal, Sobraon, and other actions in the Sikh Wars; he also served in the Indian Mutiny, the first China War, and the Bhootan campaign of 1864. In 1869, after thirty years in the Service, he retired and settled in Australia. Until a short time before his death this grand old doctor retained excellent health and full possession of his faculties: clearly he must have been a man of amazing constitution to reach so great an age after four campaigns in India and thirty years' residence in that country. Born before the first downfall of Napoleon, present at Sobraon, retired before the Franco-Prussian War, living to see the Allies well on the way to final triumph—truly a marvellous lifetime.

### RESIGNATION OF A MEDICAL SUPERINTENDENT

Dr. John Charles Keats, M.R.C.S., L.R.C.P. (Lond.), D.P.H., the medical superintendent of Camberwell Infirmary, has resigned owing to a serious breakdown in health. Dr. Keats was educated at King's College and St. Bartholomew's Hospital, where he was junior house physician, afterwards accepting a post as assistant medical superintendent at the Greenwich Infirmary. He came to Camberwell eighteen years ago, and during his long stay at the institution he successfully obtained the sympathy of the thousands of patients coming into the institution, and the admiration of his employers by his tact and courtesy. No doubt the litigation of some three years ago was a serious mental strain to Dr. Keats. It will be remembered that it was alleged Dr. Keats had whipped child patients in the infirmary so as to cause them serious bodily harm; but the actions he brought against the *London Budget* and other defendants thoroughly vindicated his character, and proved that he was a kindly, sympathetic attendant to the wants and ailments of the poor. The Local

Government Board have notified the Camberwell Board of Guardians that during the war they are not prepared to sanction the permanent appointment of any chief officer to any institution, the result being that the Board has decided to advertise for a temporary medical superintendent at a salary of £750 a year, with unfurnished house.

### CHAIRMAN'S GIFT TO MACCLESFIELD INFIRMARY.

Mr. W. Frost, chairman of the house committee of Macclesfield Infirmary, has presented to the institution the sum of £500 for the endowment of two cots. The first, in memory of his only son, it was always his wife's intention, he states, to endow. The second, Mr. Frost has endowed himself in her memory. The cots will be inscribed "William Knight Frost, 1906, given by his mother," and "Hannah Frost, 1916," respectively. In addition to holding the chairmanship of the house committee, the donor has been for many years a governor of the infirmary, in which he has always taken an active, personal interest.

### A MASONIC WAR HOSPITAL.

An interesting project for utilising the old buildings of the Chelsea Hospital for Women, in the Fulham Road, was discussed at a Mansion House meeting on June 28. It is proposed to turn it into a Freemasons' War Hospital, and after the approval of the Grand Lodge of England, of the British Red Cross Society and Order of St. John, and of the War Office was announced, it is not surprising that the resolution put to the meeting was carried unanimously. It was stated that over £2,000 had been already promised, together with a motor-car, valued at £850, given by the Hon. A. D. Ryder to be disposed of for the benefit of the hospital. The committee of the Masonic Nursing Home have obtained a short lease, with the option of purchase, of the freehold of the Chelsea Hospital now being vacated; the scheme for the nursing home is to remain in abeyance until after the war, and meanwhile fifty to sixty wounded are to be accommodated in the building. This excellent scheme does honour to all connected with it. It was indeed a happy thought on someone's part to convert the Fulham Road institution to military purposes: the buildings were erected as a hospital, and therefore the amount of adaptation required will be almost infinitesimal.

### TEXTILE SUPPLIES FOR HOSPITALS.

On page 353 of this issue of The Hospital will be found the concluding article of a series which has dealt exhaustively with the question of textile supplies for hospitals. This article, on the care of textiles, is in many ways the most valuable and instructive of the whole series, which may throughout be recommended alike to managers, housekeepers, stewards, and matrons of institutions. The subjects have been dealt with by an expert, and they are by their nature outside the ken of most of those who hold administrative appointments in hospitals, so that there is the greater need of advice and instruction about them. How markedly financial economy in hospital administration may be promoted by a knowledge of the properties of textiles is obvious from a perusal of this week's article, and the same holds good of many of its predecessors. The storage, washing, disinfection, and care generally of textile fabrics are really a scientific study more than an art; and efficiency in this respect means an enormous saving of expense to any institution. The glossary of technical terms which is appended at the conclusion of this week's article will also be found to be of very great utility to those whose duty it is to order these supplies. The subject of textile supplies may appear to the uninitiated dull; admittedly there are showier branches of hospital administration. But the conscientious official will find the problems discussed by our expert contributor to be full of interest, as well as of vital consequence to the economical working of an institution.

### THE ROYAL VICTORIA INFIRMARY, NEWCASTLE-ON-TYNE.

The annual report of this institution is of especial interest this year on the financial side, though it contains much valuable information also on other aspects of hospital administration, such as the overcoming of war-shortage difficulties. Out of a total ordinary income of £49,855 almost one-half (£24,722) comes from workmen's contributions, and £9,243 of the remainder from the War Office for treatment of military patients. The enormous sum received from the splendidly organised workpeople's funds reflects in some degree the increased prosperity of the Newcastle district from munition works; though this is to some extent offset by diminished returns from the collieries, whence there have been many enlistments. The ordinary income exceeded ordinary expenditure by £4,627, a most satisfactory result. During 1915 the managers converted over £26,000 of holdings in Consols and First War Loan into the $4\frac{1}{2}$ per cent. Loan. In order to do this it was necessary to invest £33,718 in the latter, for which purpose a loan was secured from the bankers. The result of these financial operations has been to produce an additional revenue of £186, after providing interest on the bank loan. The finance committee and the governors may be congratulated on their courage and wisdom in seizing their opportunity; as a legacy of £40,000 from Mr. J. C. Eno, who died during the year, will become payable, we may presume that the loan from the bank will be but temporary. We notice in the schedule of investments certain mortgages at $3\frac{1}{2}$ and $3\frac{3}{4}$ per cent.; unless there are valid reasons to the contrary, the finance committee might well consider the possibility of obtaining a better yield on this capital.

### ALLEGED EMBEZZLEMENT OF A LEGACY TO A HOSPITAL.

A distinctly unusual case was tried at the last Gloucester Assizes, and resulted in the acquittal of the accused. It seems that in 1892 a Miss Caroline Hughes died, leaving property worth £400 in the hands of her executors on trust to pay the income to her brother, Joseph Hughes, and after his death to pay certain legacies, one of which was £100 to the Stroud General Hospital. The legacy became payable on the death of Joseph Hughes early in 1897, and the allegation of the prosecution was that the prisoner never paid the money, but misappropriated it. The treasurer of the hospital, who is also manager of the Stroud branch of Lloyds Bank, gave evidence that the money has not been paid during his tenure of office (from 1914 onwards), and that he can find no record of its ever having been paid. The defence was that the money was paid in 1897, but the receipt for it destroyed in a fire. The judge ruled that there was no evidence of the money not having been paid. The failure of the prosecution to establish its case must be presumed to rest with the police, not with the hospital authorities; but it is distinctly unfortunate that any institution should be mixed up with a charge which cannot be sustained. The circumstances are, of course, exceptional, but the moral is clear; if a hospital were able to produce annual accounts, duly audited, kept on the Uniform System, a prosecution like this would either be successful or would not be undertaken at all.

### THE WAR AND THE OUT-PATIENT DEPARTMENT.

The movement, so long advocated in The Hospital, for making the out-patient department a consultative centre is receiving unexpected impetus from the war. In the Midlands, at all events, the giving of medicine to out-patients has been reduced very largely, and the effect of this economy may be much more than a reduction in the drug bill. It has been suggested that the new scheme may educate out-patients to use the department more as a centre for diagnosis and advice, and less as a medicine shop. This has been described by one medical man as "raising the out-patient department to the level of a consulting-room," and it will be interesting to watch the effect. No doubt much unnecessary medicine has been given by hospitals to patients who expect to receive it as a matter of course. But, while medicine is necessary, it has been found in all other centres of medical advice, inspection or consultation, that advice is not taken unless treatment is guaranteed. On the possibility of successfully referring patients to other medical men the future depends, but diagnosis and treatment are increasingly becoming complex processes for which few men in private practice have either the resources or leisure. The beginning of education, however, in this respect is certainly made in reducing or doing away with free medicine bottles.

### THE NEW WING AT THE ROYAL GWENT HOSPITAL, NEWPORT.

Lord Tredegar, on Thursday of last week, opened the new wing which has been added to the Royal Gwent Hospital, Newport, at a cost of about £14,000. By this extension an addition is made of twenty beds, bringing the total up to 175, an improved casualty ward, electrical department, and new *x*-ray room, two further operating theatres, and rooms for operating surgeons and anæsthetists. The directors estimate that the maintenance of the new wing, which has been built to the plans of Mr. H. J. Griggs, A.R.I.B.A., will necessitate an increased income of between £2,500 and £3,000 per annum. On the building a sum of £4,000 remains to be paid; the rest of the cost has been defrayed out of legacies of £5,000 each by the late Lord Tredegar and the late Mrs. Grice.

### A SPHERE OF USEFULNESS FOR THE CONSCIENTIOUS OBJECTOR.

We cannot help thinking that the Tynemouth Local Tribunal under the Military Service Acts missed an opportunity lately when applications were made for the exemption of certain Corporation employees engaged in the clearance of night-pails. It was urged that the staff of these men was already so much reduced that it is barely possible to keep the town in a sanitary condition; and the Tribunal granted conditional exemption to eight of these men. Surely this is just the sort of work to which no conscientious objector could possibly object (on grounds of conscience, at least), since it is directly concerned with the preservation of life and not at all with its destruction; if the Tribunal had sent in to the authorities a recommendation that a squad of the Non-Combatant Corps should be employed on this necessary work, we cannot imagine that the request would have been refused, and eight recruits for the Army would have been released.

### GREAT NORTHERN HOSPITAL'S DIAMOND JUBILEE.

The celebration last week of the sixtieth anniversary of the first meeting of the founders of the Great Northern Central Hospital took the form of a visit of some 500 supporters to the institution. In an informing speech Mr. G. L. Smithett, honorary secretary, recalled the opening with sixteen beds and one nurse, the purchasing of the site in 1884, and stated that 1,000 wounded soldiers had been treated. They had at present 150 beds at the War Office's disposal, and the adjoining Public Library in Manor Gardens was being prepared for the accommodation of eighty more wounded. These developments are costing more than the hospital so far has been able to raise; but the interest of Islington people, shown by the large attendance, should result in the necessary effort being made to provide the money required.

### GARDEN PARTIES FOR THE WOUNDED.

The Birmingham branch of the British Red Cross Society has been appealing for subscriptions towards a fund to enable it to hire motors and to run the car lately given to it, with the object of taking wounded men to garden parties organised on their behalf. During June the branch was holding these parties several days a week, and they have been locally organised on a large scale. The numbers entertained have varied from twenty-five to 1,000. Apparently this form of entertainment, so agreeable in fine weather, has been more completely arranged in the Birmingham district than elsewhere. Men in the big hospitals may be well catered for in the matter of the wounded, but we commend the scheme to those in charge of convalescent homes for soldiers. In these places, because the numbers are small, entertainments are somewhat haphazard; but as the homes are always in country districts, where houses with large gardens usually abound, the organisation of garden parties would be a welcome and practicable way of providing a much-appreciated and inexpensive recreation.

### A DOG ON THE STRENGTH.

The war has led to the relaxation of many hospital rules, and the latest reported instance is that whereby the dog "Rara," a Great Dane, the mascot of the Auckland (New Zealand) Mounted Rifles, has become attached to the 4th London General Hospital. The story goes that one of the men in the regiment made a great pet of "Rara," who, on her friend becoming a patient, and consequently an absentee, lost her appetite and became dispirited. On hearing the news the man in his turn became worried, and, for the sake of both patients, the dog was brought to the hospital, where she is understood to be wearing an official identification badge fastened to a chain, provided at the expense of the regiment. It is perhaps fortunate that one mascot is usually sufficient for each regiment, for a vista of hospital wards in which patients were surrounded by the kennels and cages of their pets would make a strait-laced staff, if any remains, faint with disapproval.

### TRIBUTE TO A HOSPITAL SECRETARY.

Not for the first time, if we remember, Mr. Tom Morgan, secretary of the Pontypool and District Hospital, has received the formal thanks of his board for satisfactory or exceptional work. The Chairman, Mr. Benjamin Nicholas, lately referred to the extraordinary duties which had fallen to the secretary in connection with the arrangements for the Belgian soldiers who were accommodated in the hospital. In connection with the building of the Nurses' Home and, we understand, the workpeople's collections, the secretary has also worked with energy and success. A tribute of this kind earned as the result of complex activities in difficult times is evidence of keenness and hard work.

### AN ENDOWED DISPENSARY FOR DUBLIN.

The Courts have confirmed the provisions of the will of the late Mr. Charles Lawler, formerly proprietor of the Imperial Hotel, Dublin, whereby a sum of £15,000 was set aside to build and equip a suitable dispensary to be attached to St. Vincent's Hospital, St. Stephen's Green, Dublin, for the provision of free medical advice and relief to the poor. The testator also bequeathed the residue of his property to the legatees mentioned in his will and codicil in proportion to the amounts left respectively to them. On July 15, 1915, the Court ordered the distributive share under the residuary bequest to be added to the above sum of £15,000. Counsel, on behalf of the Trustees of St. Vincent's Hospital, lately declared in the Chancery Division that, owing to the residue of the estate being very large, a sum of £30,000 was now available for the dispensary. The scheme provides for the dispensary to be attached to St. Vincent's Hospital and vested in the Sisters of Charity or in the medical and surgical staff acting under their authority. The unexpended balance of the trust funds will be invested and the proceeds devoted to the management and maintenance of the institution. In accordance with the wishes of the testator, the new dispensary will be dedicated to him, and a tablet recording his gift is to be placed in the building.

### "GAZETTES" FOR SANATORIA.

We have received the first number of the *Cranham Lodge Sanatorium Gazette*, an arrival which seems to show that the success which has attended the war hospital publications is proving infectious. A sheet of eight pages, it is mostly given up to brief paragraphs on institutional points of local interest, changes of rules, notes on new arrivals, and a certain amount of chaff. A good point is made in appealing to the patients to send to the editor any complaints or grievances; but it is to be hoped that they will find other topics out of their own experiences and interests on which to spend their talents. Sanatorium patients are often old stagers. They have been examined by numerous doctors in England and perhaps abroad; in some cases they have had experience of a variety of institutions. The impression this has left upon their minds is naturally interesting and worth recording. Such a *Gazette* as this should give these records to the world. For instance, one old stager told us that experience had convinced him that a doctor's view of smoking depended in the main on whether he was a smoker himself; and that from the way in which a man listened to his chest he could always tell whether he knew his work or not. As he had been ill for eleven years at the time he made this statement the remark, whether true or false, was certainly based upon experience. We give these two minor examples for what they may be worth in suggesting topics of interest to be examined in this and any other sanatorium *Gazettes*. The great thing, however, is to make the standard as high as possible. Nothing is so dull as a silly joke or pseudo-comic story.

### A WELSH BARD IN THE R.A.M.C.

The fortnightly *Gazette* of the 1st Eastern General Hospital, Cambridge, in its second June number, had the happy thought to reprint Mr. Rudyard Kipling's poem "Kitchener's School" as a memorial and epitaph to the late Field-Marshal. The whole number is rich in verse, and certainly few poems in *Gazettes* approach in vigour and swing the "Story of the Great War," written by Private Alfred Jenkins, 2/1st W.C.C.S., R.A.M.C.; it is hardly surprising to learn that the "story" was the chair poem at Bedford Divisional Eisteddfod on Easter Monday. The number also contains a letter from Captain Corfield, under whom a sanitary party from the hospital was taken to Gallipoli last year, recording the specially good work of Staff-Sergeant A. C. Read and Sergeant Miller. There is also quoted from the 4th Southern Hospital *Gazette* "A True Story" that might have come out of the pages of Dickens.

### CONSUMPTION AND THE BOOT TRADE.

The question of the prevalence of phthisis among workers in the boot and shoe industry was discussed at the recent conference of the National Union of Boot and Shoe Operatives. The rate of mortality from tuberculous disease among boot-workers is admittedly far too high, but there is no reason to suppose that it exceeds that met with in other occupations in which dust is an important factor. The statement made by one of the speakers that consumption is "characteristic of the boot trade" strikes us as being too sweeping, for it must be borne in mind that the modern factory with its well-ventilated rooms is infinitely superior to the close workshops of half a century ago. However well constructed a building may be, the workers who are employed therein cannot be expected to remain free from tuberculosis if they habitually neglect the rules and sanitary precautions made for their safety by those who have special knowledge of the best methods for its prevention. No relaxation in the inspection of all such factories and workshops should be countenanced at the present time, and penalties should be strictly enforced in cases of non-observance of rules, especially that against spitting. We notice that a subcommittee of the Union has been formed to consider the question of sanatorium provision and Government co-operation in connection therewith.

### A WAR VACANCY AT WITHINGTON.

When a recommendation was brought forward at last week's meeting of the Manchester Board of Guardians that Dr. Gamble, the present temporary assistant medical officer at Withington institution, should be appointed the temporary medical officer owing to the death of Dr. Austen, some opposition was raised, and an amendment was moved that the vacancy be advertised. It was urged in favour of the appointment that it was only a temporary one, that the military authorities, who are in possession of Withington, were anxious for it to be filled, and that the reason the vacancy was not being advertised was that the Local Government Board would not allow the appointment of a man of military age, although it would be inadvisable to have as medical officer an "old man past military age." The upshot was that the amendment, for which eleven votes were cast, was lost, and the appointment of Dr. Gamble agreed to.

### TUBERCULOSIS AND THE WAR.

That portion of the Report of the Local Government Board for 1914-15, recently issued, which is occupied with the tuberculosis campaign shows that, although some of the work has been severely handicapped in consequence of the war, progress has been made in many directions and in different parts of the country. In some instances where tuberculosis hospitals have been requisitioned by the military authorities, other buildings have been found and equipped to meet the exigencies of the case. The absence of nearly one-third of the responsible clinical officers has necessitated special arrangements being made, such as securing the services of women doctors or of retired practitioners, while many tuberculosis officers have undertaken extra duties in order that the work of fighting consumption in some outlying district might not languish. The willingness of the medical profession to serve their country in so many different ways has not failed to impress itself upon Mr. Long, who is to be congratulated upon the courageous way in which he has handled one of the most difficult problems of these abnormal times.

### THE PORTER'S BEER.

An old custom, that may be regarded as doomed, has now been discarded at Worcester Infirmary—that, namely, of supplying the head porter and his assistant with beer. The change made last month took the form of the following resolution, which was agreed to on the recommendation of the house committee: "That increases of salary be given to the head porter (W. Bateman) and the assistant porter (W. J. Vaughan), the former to have £45 a year instead of £40, and the latter £24 instead of £20." Everything is found for both officers, including one suit of uniform, except beer. Mr. Park, who presided at the infirmary committee meeting, said that it was no longer the custom to supply the staff of institutions with beer, and he proposed its discontinuance. In reply to questions, Mr. Park said that the committee preferred to regard the increase as not in lieu of beer, though that is what it amounts to. The discussion is worth recording as a minor event in hospital history—the passing of the porter's beer, an event at ne time that would have sounded incredible.

### THIS WEEK'S DRUG MARKET.

Business continues rather quiet and price changes have not been numerous. An easier tendency in prices is again noticeable, and buyers would be well advised to exercise caution in the purchase of those drugs that are quoted at extraordinary figures; they should limit their purchases to their immediate requirements. There has been no improvement in the demand for bromides, but the market is slightly steadier; salicylates still have a slightly downward' tendency in price. Phenolphthalein seems to be quite unobtainable, the last available having been sold at an extraordinary price. Cod-liver oil is cheaper, but still far too dear to attract business. Acetanilide is distinctly lower in price; borax and boric acid are dearer. Further business has been done in Japanese refined camphor at a rather higher price. At the recent public sale of drugs in Mincing Lane the demand was very quiet, but there were few price changes of particular note, senna-leaves and honey, however, had a lower tendency.

